# Different Synaptic Plasticity After Physiological and Psychological Stress in the Anterior Insular Cortex in an Observational Fear Mouse Model

**DOI:** 10.3389/fnsyn.2022.851015

**Published:** 2022-05-11

**Authors:** Wenlong Shi, Yuan Fu, Tianyao Shi, Wenxia Zhou

**Affiliations:** ^1^State Key Laboratory of Toxicology and Medical Countermeasures, Beijing Institute of Pharmacology and Toxicology, Beijing, China; ^2^Nanjing University of Chinese Medicine, Nanjing, China

**Keywords:** psychological stress, physiological stress, anterior insular cortex, long-term potentiation, long-term depression, multielectrode array

## Abstract

Post-traumatic stress disorder (PTSD) can be triggered not only in people who have personally experienced traumatic events but also in those who witness them. Physiological and psychological stress can have different effects on neural activity, but little is known about the underlying mechanisms. There is ample evidence that the insular cortex, especially the anterior insular cortex (aIC), is critical to both the sensory and emotional experience of pain. It is therefore worthwhile to explore the effects of direct and indirect stress on the synaptic plasticity of the aIC. Here, we used a mouse model of observational fear to mimic direct suffering (Demonstrator, DM) and witnessing (Observer, OB) of traumatic events. After observational fear training, using a 64-channel recording system, we showed that both DM and OB mice exhibited a decreased ratio of paired-pulse with intervals of 50 ms in the superficial layers of the aIC but not in the deep layers. We found that theta-burst stimulation (TBS)–induced long-term potentiation (LTP) in OB mice was significantly higher than in DM mice, and the recruitment of synaptic responses occurred only in OB mice. Compared with naive mice, OB mice showed stronger recruitment and higher amplitude in the superficial layers of the aIC. We also used low-frequency stimulation (LFS) to induce long-term depression (LTD). OB mice showed greater LTD in both the superficial and deep layers of the aIC than naive mice, but no significant difference was found between OB and DM mice. These results provide insights into the changes in synaptic plasticity in the aIC after physiological and psychological stress, and suggest that different types of stress may have different mechanisms. Furthermore, identification of the possible causes of the differences in stress could help treat stress-related disorders.

## Introduction

Post-traumatic stress disorder (PTSD) is a serious psychiatric disorder that can occur in people who have experienced or witnessed a traumatic event. The increasing prevalence of PTSD has been associated with rising social and economic costs (Schäfer and Fisher, 2011; Maren and Holmes, 2016). In recent years, much progress has been made in understanding the mechanisms of PTSD using animal models such as fear, single prolonged stress, or restraint stress (Kavushansky et al., 2009; Comeras et al., 2021). In these models, the effect of psychological stress as one factor among several stressors cannot be studied separately (Lesnikova et al., 2021). Some studies suggest that there may be a different neural mechanism between psychological and physiological stress. The observational fear learning (OFL) paradigm is the most commonly used animal model to study empathic fear (Panksepp and Lahvis, 2011; Keum et al., 2016; Kim et al., 2019). Observer mice were exposed to psychological stress only, whereas demonstrator mice underwent a direct shock experience. Using this model, we can examine the different mechanisms of these two types of stress in mice.

The insular cortex (IC) is a complex and richly interconnected structure that receives afferent projections from thalamic nuclei, and forms an affective pain system with the amygdala, limbic system, and cortical association areas (Craig et al., 2000; Craig, 2014), positioning it as a site of multisensory integration (Gogolla, 2017). Direct electrical stimulation of the IC can elicit painful and somatic sensations in humans, supporting its critical role in pain and sensory perception. The accumulated evidence suggests that the IC is a cortical node associated with the integration of sensory input and emotion. Damage to the IC results in patients feeling less pain or empathy for pain (Benarroch, 2019). In particular, the anterior insular cortex (aIC), which mediates interoceptive attention, is thought to be associated with emotional awareness (Craig, 2009; Shi et al., 2018), and many reports suggest that the aIC is necessary for empathic pain perception (Gu et al., 2013; Abu-Akel et al., 2015). Therefore, it is worthwhile to establish whether the aIC plays a different role in psychological and physiological stress in animal models.

Synaptic plasticity is the core mechanism of PTSD and the most important issue in the treatment of this disease (Zhang and Bramham, 2020). Long-term potentiation (LTP) of synaptic transmission is the major form of activity-dependent plasticity in the central nervous system (CNS) and a key synaptic model for investigating the cellular and molecular mechanisms of chronic pain and anxiety (Liu et al., 2013a). Long-term depression (LTD) is another important form of synaptic plasticity in the CNS (Collingridge et al., 2010). LTP and LTD are widespread phenomena that occur at excitatory synapses in the brain and demonstrate the ability of synaptic connections between neurons to be weakened or strengthened (Malenka and Bear, 2004). In general, disruption of synaptic plasticity has been implicated in CNS disorders, from neurodegenerative disorders to stress-related trauma (He et al., 2018). However, little is known about the cellular mechanisms in the aIC which may differ between psychological and physiological stress. Understanding the cellular and molecular mechanisms at central synapses may help us uncover the differential impact of these two types of stress on brain functions.

In this study, we generated a new paradigm of observational fear learning (OFL) to investigate stress-induced neuronal plasticity at the synaptic level using a 64-channel multielectrode dish recording system, and found that psychological and physiological stress led to layer-related differences in synaptic transmission and plasticity in the aIC.

## Materials and Methods

### Animals

Adult male C57BL/6J mice (12–13 weeks old) were used for this experiment. All animals were socially housed in a room with a 12:12 h light and dark cycle (lights on at 7:00 a.m.) at 25°C and received water and food *ad libitum*, except during behavioral testing. All research protocols conformed to the National Institute of Health guidelines and were approved by the Animal Care and Use Committee of the Beijing Institute of Pharmacology and Toxicology.

### Observational Fear Learning Model

Mice were first acclimated to the chamber (conditioning cage), a behavioral testing device (400 mm × 400 mm × 400 mm) with a transparent cylinder in the corner. Before the conditioning session, the test cage was wiped with 70% ethanol. In this conditioning system, two male C57BL/6J mice that had previously been housed together for 5 weeks were placed individually in the chambers of the observational fear-conditioning apparatus separated by a transparent plexiglass cylinder, and one mouse (observer) was allowed to observe the other (demonstrator). After a 4-min interaction period, the demonstrator mouse was administered a 2-s foot electric shock (1 mA) every 10 s for 4 min. To assess retrieval memory, observer mice were placed back into the chamber 24 h after the 4-min training (Keum et al., 2018). The behavior of the mice was recorded using Any–Maze software (Stoelting Co., Chicago, United States). Motionless bouts lasting longer than 500 ms were considered freezes.

### The Multi-Channel Probe Preparation

The 64-channel multielectrode array recording system (MED64; Panasonic Alpha-Med Sciences, Osaka, Japan) was used for extracellular field potential recordings. The MED64 dish (P515A, Panasonic, Japan) has an array of 64 square planar microelectrodes, each 50 μm × 50 μm in size, arranged in an 8×8 pattern, with a distance of 150 μm. Before use, the surface of the MED64 probe was treated with 0.1% polyethyleneimine (Sigma, St. Louis, MO, United States; P-3143) in 25 mmol/L borate buffer (pH 8.4) overnight at room temperature according to previously reported protocols. The surface of the probe was then flushed three times with sterile distilled water to remove all residues (Liu et al., 2013b).

### Brain Slice Preparation

The rostrocaudal levels corresponded to 0.9–1.7 mm aIC relative to the bregma (Shi et al., 2018). Adult mice were anesthetized with gaseous isoflurane, and brains were removed. Coronal brain slices (300 μm) containing the aIC were prepared in ice-cold oxygenated (95% O_2_ and 5% CO_2_) artificial cerebrospinal fluid (ACSF) (in mM:124 NaCl, 2.5 KCl, 1.0 NaH2PO4, 1 MgSO4, 2 CaCl_2_, 25 NaHCO_3_, and 10 glucose, pH 7.35–7.45). For electrophysiological recordings, sections were transferred to a recovery chamber containing oxygenated (95% O_2_ and 5% CO_2_) ACSF at 30–32°C for at least 1–2 h (Liu et al., 2013a).

### Field Potential Recording in Insular Cortex Slices

After incubation, a slice containing the aIC was positioned on the MED64 probe so that the different aIC layers covered most of the 64 electrodes. Then a fine-mesh anchor (Warner Instruments, Harvard) was carefully placed on the slice and the slice was continuously perfused with oxygenated fresh ACSF at a rate of 2–3 ml/min throughout electrophysiological recording.

After a 15-min recovery period, the stimulation site was placed in the deep layers IV–V of the aIC, which can elicit the best synaptic responses from deep to superficial layers. A biphasic constant-current pulse stimulation (0.2 ms duration) generated by the data acquisition software (Mobius, Panasonic Alpha-Med Sciences) was applied to the channel, and the intensity was adjusted to elicit 40–60% of the maximum slope of the excitatory postsynaptic potential (fEPSP) near the stimulation site.

The channels with fEPSP and amplitude above 10 μV were defined as activated channels, and their responses were sampled every 0.5 min. Baseline responses were first recorded until the variation was <5% in most active channels within 15 min. Then, a TBS protocol (4 pulses at 100 Hz for each burst) was applied to induce LTP. For LTD induction, a stable baseline (as for LTP recording) was recorded for 15 min and then a classical LFS protocol (1 Hz, 900 pulses) was performed as previously reported (Liu et al., 2013a; Liu et al., 2020). When the number of unstable channels was >10%, the slice was not considered. The slope was normalized as a percentage of the baseline level. For comparison of LTP and LTD, the average fEPSP slope of the last 10 min recordings was statistically compared between naive, observer, and demonstrator mice. For the paired-pulse ratio (PPR), the ratio of the slope of the second response to the slope of the first response was calculated and averaged. The interval varied between 25, 50, 75, and 100 ms (Bornschein et al., 2013).

### Data Analysis

Data are presented as mean ± SEM. Statistical comparisons between two groups were performed using the unpaired Student’s *t*-test with Welch’s correction, one-tailed ANOVA followed by Tukey’s multiple comparison test. Statistical analyses between multiple groups were performed using two-way ANOVA followed by Sidak’s multiple comparison test and Tukey’s multiple comparison test (GraphPad Prism 8.0.1), to identify significant differences. In all cases, *p* < 0.05 was considered statistically significant.

## Results

### Psychological Stress-Related Behavior in the Observational Fear Model

**FIGURE 1:**
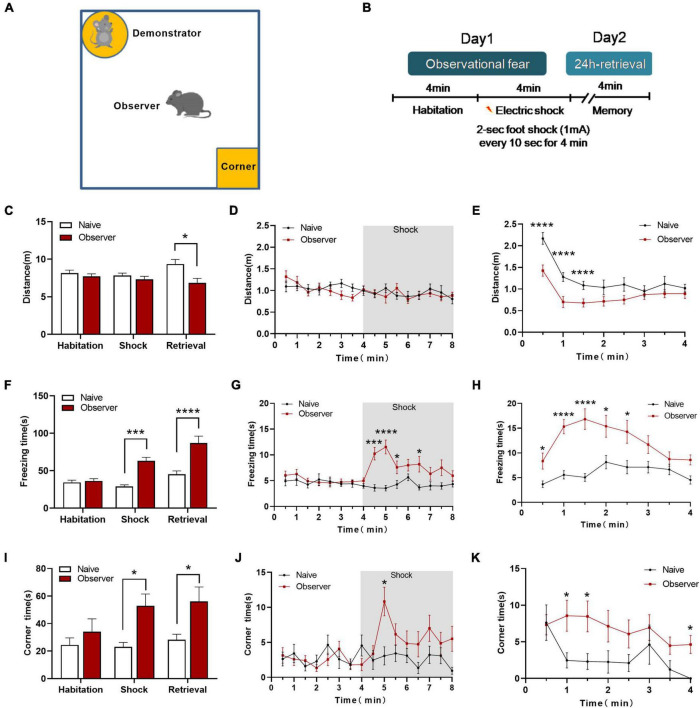
The psychological stress-related behaviors of observational fear learning model. **(A)** Diagram of the observational fear chamber; **(B)** Outline of the observational fear behavioral paradigm; **(C)** Distance moved in the chamber of naïve and observer mice during three phases. **(D)** The variation of distance during Day 1. **(E)** The measurement of distance during 24-h retrieval phase. **(F)** Vicarious freezing of naïve and observer mice during three phases. **(G)** The variation of vicarious freezing time during Day 1. **(H)** The measurement of freezing time during 24-h retrieval phase. **(I)** Time spent in the corner zone of naïve and observer mice during three phases. **(J)** The variation of corner time during Day 1. **(K)** The measurement of corner time during 24-h retrieval phase; Data are shown as mean ± SEM (naïve *n* = 13, observer *n* = 16) and compared by two-way ANOVA analysis followed by Sidak’s multiple comparisons test, *p < 0.05, ***p < 0.001, ****p < 0.0001, vs. naïve.

Translational rodent models of emotion that capture aspects of social affect, including emotional stress and social buffering, should reveal social perception and integrated social cognitive processes (Olsson and Phelps, 2007; Meyza et al., 2017). However, previous models assessed observational fear only by vicarious freezing, making it difficult to identify the comprehensive psychological stress-related emotions without physiological pain or discomfort. We developed a device for measuring observational fear to assess psychological stress-related emotions (Figure 1A). In our paradigm, the observer mouse without prior aversive experience (electric foot shock) is stressed for context-dependent fear by observing the demonstrator mouse receiving electric foot shocks (physiological stress). In the habituation phase, the observer mouse is allowed to interact with the demonstrator mouse, which is placed in the cylinder without being shocked. In the electric shock phase, the demonstrator mouse receives a 2 s foot-shock every 10 s for 4 min while the observer mouse watches. Twenty-four hours later, in the memory phase, the observer mouse is returned to the same chamber alone for 4 min (Figure 1B). During the memory phase, the observer mice showed a significant deficit in locomotion [shock phase: *t*_(27)_ = 0.984, *p* = 0.334, memory phase: *t*_(27)_ = 2.902, *p* = 0.017, unpaired *t*-test] (Figures 1C–E). The observer mice exhibited significant freezing behavior during the shock and memory phase [shock phase: *t*_(27)_ = 3.745, *p* = 0.0009, memory phase: *t*_(27)_ = 6.074, *p* = 0.0001, unpaired *t*-test] compared to naive mice (not exposed to shock demonstration) (Figures 1F–H). This increased vicarious fear response also correlated strongly with the change in avoidance behavior [Shock phase: *t*_(27)_ = 3.004, *p* = 0.015 memory phase: *t*_(27)_ = 2.293, *p* = 0.029, unpaired *t*-test] (Figures 1I–K). This novel observational fear monitoring device enables the detection of psychological stress in mice, manifested as freezing, avoidance, and escape behavior when they observe the distress of another mouse. Although we did not analyze the behavior of demonstrator mice, many reports using a variety of behavioral tests suggested that electric foot shocks induce mental disorders (Bali and Jaggi, 2015; Kaur et al., 2015).

### Presynaptic Transmission in Different Layers of the Anterior Insular Cortex in Observer and Demonstrator Mice

**FIGURE 2:**
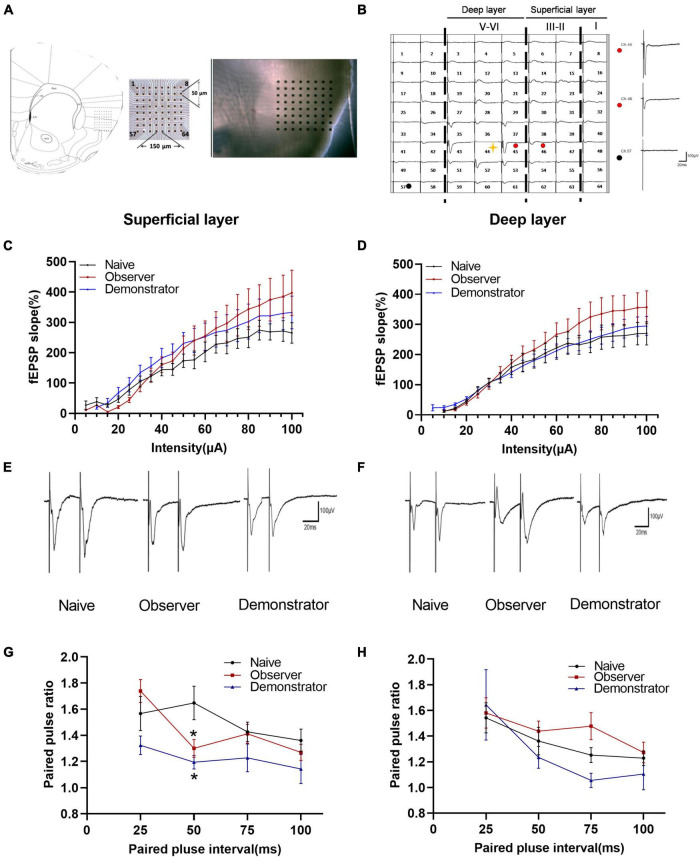
Changed presynaptic transmitter release probably within aIC of observer and demonstrator mice. **(A)** Left: schematic diagram showing location of the MED64 probe on the coronal IC slice; Right: light microscopy photograph showing relative location of aIC within the probe; **(B)** Spatial distribution of extracellular field potential induced by electrical stimulation on channel 45 (marked as red circle) in layers VI of aIC; **(C,D)** Input–output curve of fEPSP slope (%) vs. stimulus intensity (mA) in the slice among naïve (*n* = 4 slices of 3 mice), observer (*n* = 4 slices of 3 mice), and demonstrator (*n* = 7 slices of 5 mice) in the superficial **(C)** and in the deep layers **(D)** of aIC; **(E,F)** Example traces of paired-pulse facilitation (PPF) with an interval of 25 ms recorded in the superficial **(E)** and deep layers **(F)** of aIC; **(G,H)** The paired-pulse ratios (slope of fEPSP2/slope of fEPSP1) recorded with intervals of 25, 50, 75, and 100 ms in superficial **(G)** and deep layers **(H)** of aIC; Data are shown as mean ± SEM (PPF: Naïve *n* = 4 slices of 3 mice, observer *n* = 7 slices of 6 mice, and demonstrator *n* = 4 slices of 4 mice) and compared by two-way ANOVA analysis followed by Tukey’s multiple comparisons test, *p < 0.05, vs. naïve.

The aIC is involved in the appraisal and experience of emotion and interoceptive perception and is activated both during self-experienced pain and during the observation of pain (Craig, 2009; Singer et al., 2009; Lamm et al., 2011; Peltz et al., 2011; Gu et al., 2013). We used a 64-channel multielectrode array to record the spatial and temporal distribution of extracellular field responses in the aIC of adult mice (Figure 2A). The stimulation site was located in the deep layers (layers V–VI) of the aIC. As a representative example, activated channels were recorded from the superficial to the deep layer (Figure 2B). To investigate whether basal glutamatergic synaptic transmission was altered by observational fear, stimulus–response relationships for fEPSPs (input–output curve) from naive, observer, and demonstrator mice were compared. There were no significant differences between the input–output curves of each group in superficial/deep layers [Naïve *n* = 4, Observer *n* = 4, Demonstrator *n* = 7, superficial layer: *F*_(2_,_56)_ = 1.009, *p* = 0.371, deep layer: *F*_(2_,_55)_ = 1.061, *p* = 0.353, one-way ANOVA] (Figures 2C,D). To check whether there were changes in presynaptic transmission, paired-pulse facilitation (PPF) was recorded. In the superficial layers of the aIC, the paired-pulse ratio (PPR) was significantly decreased at time intervals of 50 ms in observer and demonstrator mice compared with naive mice [*F*_(2_,_183)_ = 5.256, *p* = 0.006, vs. naïve, *p* = 0.013 and *p* = 0.019 at 50-ms interval for observer and demonstrator mice, respectively; two-way ANOVA analysis followed by Tukey’s multiple comparison test, *n* = 4–7 slices/4–6 mice] (Figures 2E,G). No significant differences were measured in PPR at different intervals in the deep layers [*F*_(2_,_172)_ = 1.643, *p* = 0.196, vs. naïve, *p* = 0.850 and *p* = 0.829 at 50-ms interval for observer and demonstrator mice, respectively] (Figures 2F,H). These data suggest that presynaptic transmission was increased in the superficial layers of the aIC after observational fear and electric foot shock.

### Weak Induction of Long-Term Potentiation in Observer but the Loss in Demonstrator Mice

**FIGURE 3:**
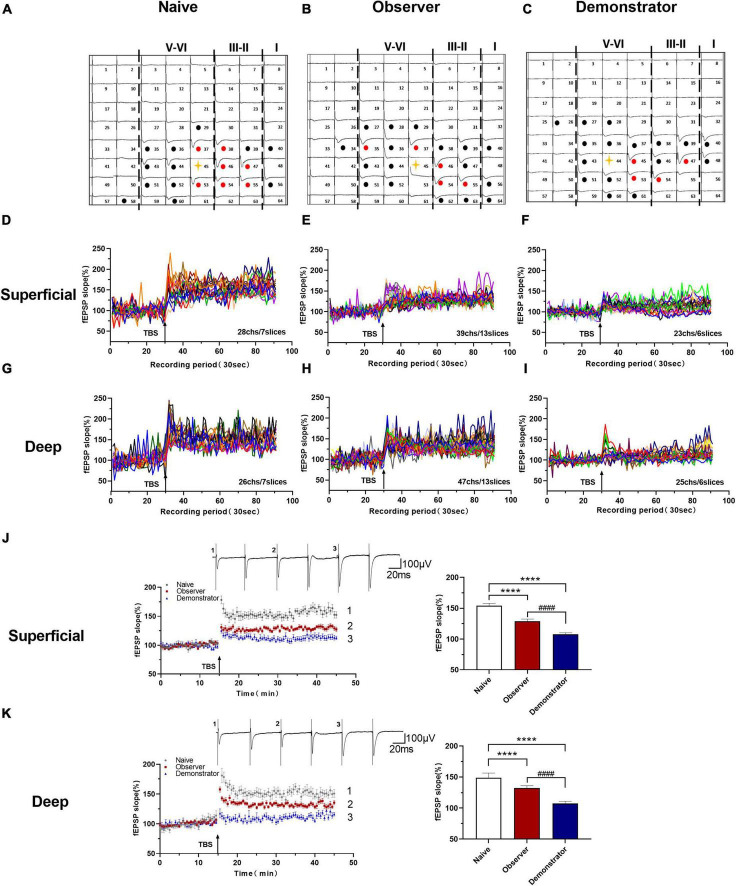
Time course LTP in the aIC of observer and demonstrator mice. **(A–C)** Samples of an overview of multisite synaptic responses recorded at baseline (black) and showing LTP after TBS (red) in naïve **(A)**, observer **(B)**, and demonstrator mice **(C)**, respectively. The flash denotes the stimulated channel, red- and black-filled circles mark all activated channels, vertical lines demarcate different layers; **(D–I)** All channels with LTP of naïve, observer, and demonstrator mice. **(D)** 28 channels of 7 slices with LTP in the superficial layers of aIC in naïve mice. **(E)** 39 channels of 13 slices with LTP in the superficial layers of aIC in observer mice. **(F)** 23 channels of 6 slices with LTP in the superficial layers of aIC in demonstrator mice. **(G)** 26 channels of 7 slices with LTP in the deep layers of aIC in naïve mice. **(H)** 47 channels of 13 slices with LTP in the deep layers of aIC in observer mice. **(I)** 25 channels of 6 slices with LTP in the deep layers of aIC in demonstrator mice; **(J,K)** Left: Time course of averaged fEPSP slope of all active channels from the superficial **(J)** and deep layers **(K)** of aIC in naïve, observer, and demonstrator mice. The arrow indicates the time of TBS application in the deep layer V/VI. Right: the average slope and of all active channels within the last 10 min recording in the superficial **(J)** and deep layers **(K)** of aIC; Data are shown as mean ± SEM (Naïve *n* = 7 slices of 6 mice, observer *n* = 13 slices of 9 mice, and demonstrator *n* = 6 slices of 6 mice) and compared by unpaired *t*-test, ****p < 0.0001, vs. naïve; ^####^*p* < 0.0001, observer vs. demonstrator.

Long-term potentiation is a primary experimental model for chronic pain and anxiety-related synaptic changes (Bliss and Collingridge, 1993; Zhuo, 2008). LTP is sensitive to stress, especially inescapable and non-escapable stress (Richter-Levin and Xu, 2018). We successfully induced LTP in both superficial (layers II–III) and deep layers (layers V–VI) around the stimulation site (Ch. 44/45) after TBS in slices from naive, observer, and demonstrator mice using the MED64 recording system (Figures 3A–C), as previously described (Liu et al., 2013a,b). In 7 slices of the aIC from naive mice, 28 channels and 26 channels showed LTP in the superficial and deep layers, respectively (Figures 3D,G). In 13 slices of the aIC from observer mice, 39 channels and 47 channels showed LTP in the superficial and deep layers, respectively (Figures 3E,H). In 6 slices from demonstrator mice, 23 channels and 25 channels showed LTP induction in the superficial and deep layers, respectively (Figures 3F,I). aIC slices (both superficial and deep layers) from shocked demonstrator mice, did not show induction of LTP [Slope: 107.795 ± 5.384% of baseline, *t*_(41)_ = 47.240, *p* < 0.0001 and 107.558 ± 4.469% of baseline, *t*_(42)_ = 23.850, *p* < 0.0001 for superficial and deep layers, respectively, *n* = 6 slices/6 mice; vs. naïve, *n* = 6 slices/6 mice, unpaired *t*-test]. LTP was significantly reduced in slices from the aIC of observer mice [Slope: 154.145 ± 3.358% of baseline in naïve and 128.799 ± 3.519% in observer for superficial layers, *t*_(40)_ = 22.190, *p* < 0.0001; Slope: 148.872 ± 9.763% in naïve and 132.249 ± 4.581% for deep layers, *t*_(42)_ = 9.487, *p* < 0.0001. Superficial layer: *t*_(41)_ = 22.370, *p* < 0.0001 and deep layer: *t*_(43)_ = 2.194, *p* < 0.0001, observer vs. demonstrator, unpaired *t*-test, *n* = 7 slices/6 mice, observer *n* = 13 slices/13 mice] (Figures 3J,K). These results suggest that synaptic responses in the aIC are stronger after physiological stress than after psychological distress.

**FIGURE 4:**
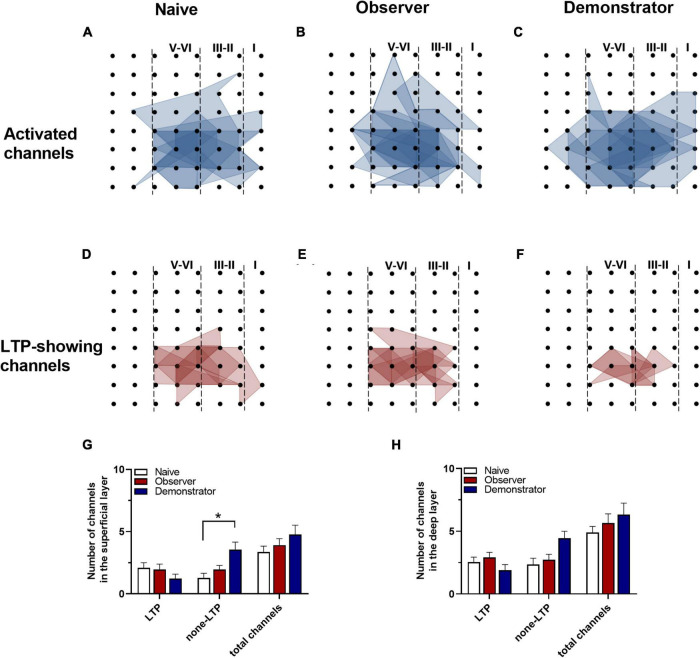
Spatial analysis on LTP distribution in aIC. **(A–F)** The polygonal diagram of activated (blue) and LTD-showing (red) channels within aIC in naïve **(A,D)**, observer **(B,E)**, and demonstrator mice **(C,F)**. **(G,H)** The average number of all channels of naïve, observer, and demonstrator mice in the superficial **(G)** and deep layers **(H)**. Data are shown as mean ± SEM (Naïve *n* = 7 slices of 6 mice, observer *n* = 13 slices of 9 mice, and demonstrator *n* = 6 slices of 6 mice) and compared by two-way ANOVA analysis followed by unpaired *t*-test,*p < 0.05, vs. naïve.

To perform the LTP across an extended space scale, we applied the previous method (Liu et al., 2013a). The blue represents the activated channels and the red denotes the LTP-occurring channels in the spatial characteristics of aIC, which is distinguishable between superficial and deep layers. Among all the slices, the tendency in the shrinkage of LTP map was similar to the potentiation plasticity with that of electrically induced fEPSP slope (Figures 4A–F). Although there was a significant difference in the number of channels without potentiation between naive and demonstrator mice [Superficial layer: 2.091 ± 0.415 channels with LTP and 1.273 ± 0.384 channels with none-LTP in naive, 1.962 ± 0.435 channels with LTP and 1.962 ± 0.326 channels with none-LTP in observer, 1.222 ± 0.364 channels with LTP and 3.556 ± 0.603 channels with none-LTP in demonstrator in each slice of mice on average, LTP channels: *t*_(35)_ = 0.178, *p* = 0.859, naive vs. observer; *t*_(33)_ = 0.954, *p* = 0.347, demonstrator vs. observer; *t*_(18)_ = 1.537, *p* = 0.142, demonstrator vs. naïve. None-LTP channels: *t*_(35)_ = 1.228, *p* = 0.227, naive vs. observer, *t*_(33)_ = 2.427, *p* = 0.209, demonstrator vs. observer; *t*_(18)_ = 3.310, *p* = 0.014, demonstrator vs. naïve, unpaired *t*-test, naïve *n* = 7 slices/6 mice, observer *n* = 13 slices/13 mice, demonstrator *n* = 6 slices/6 mice], no statistical difference in the number of channels showing LTP in the spatial analysis of post-LTP distribution was found [Deep layer: 2.545 ± 0.390 channels with LTP and 2.364 ± 0.491 channels with none-LTP in naive, 2.923 ± 0.400 channels with LTP and 2.731 ± 0.439 channels with none-LTP in observer, 1.889 ± 0.455 channels with LTP and 4.444 ± 0.556 channels with none-LTP in demonstrator in each slice of mice on average, LTP channels: *t*_(35)_ = 0.566, *p* = 0.575, naive vs. observer; *t*_(33)_ = 1.410, *p* = 0.168, demonstrator vs. observer; *t*_(18)_ = 1.102, *p* = 0.285, demonstrator vs. naïve, none-LTP channels: *t*_(35)_ = 0.493, *p* = 0.627, naive vs. observer; *t*_(33)_ = 2.097, *p* = 0.054, demonstrator vs. observer; *t*_(18)_ = 2.813, *p* = 0.115 demonstrator vs. naïve, unpaired *t*-test] (Figures 4G,H). Therefore, unlike psychological stress, the effects of physiological stress on LTP have both temporal and spatial aspects.

### Recruited Responses Are Elicited After Theta-Burst Stimulation in Observer but Not Demonstrator Mice

**FIGURE 5:**
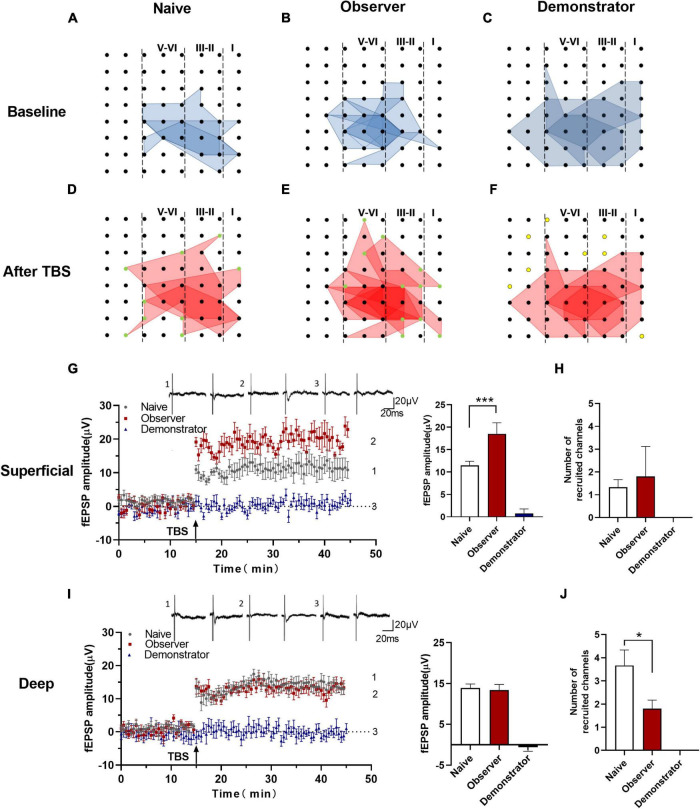
Recruited responses in the aIC of observer after TBS. **(A–F)** The network propagation of synaptic responses in aIC after TBS of naïve **(A,D)**, observer **(B,E)**, and demonstrator mice **(C,F)**. Basal activated areas (blue), and recruited areas (red), and the recruited channels are shown as green spot, channels out of the edge of the activated channels in demonstrator mice are shown as yellow spot; **(G,I)** Left: Time course of averaged fEPSP amplitude of recruited channels from the superficial **(G)** and deep layers **(I)** in naïve and observer mice and the no responses channels in demonstrator mice. The arrow indicates the time of TBS application in the deep layer V/VI; Right: the average fEPSP amplitude of the channels within the last 10 min recording in the superficial **(G)** and deep layers **(I)**; **(H,J)** Average number of channels of naïve and observer in the superficial **(H)** and deep layers **(J)**. Data are shown as mean ± SEM (naïve *n* = 3 slices of 3 mice, observer *n* = 6 slices of 6 mice, and demonstrator *n* = 4 slices of 4 mice) and compared by unpaired *t*-test with Welch’s correction, *p < 0.05, ***p < 0.001, vs. naïve.

One of the advantages of multichannel recording is that it allows observing the recruitment of channels that are initially inactive but can be recruited by TBS induction. Previous research indicates that some silent responses are converted to non-silent responses after LTP induction (Song et al., 2017). Consistent with previous studies, the recruited channels were mainly at the edge of the activated area (Chen et al., 2014b), and the amplitude, which was approximately 0 μV at baseline, increased with time after applying the TBS protocol. We analyzed all activated channels after TBS in naive, observer and demonstrator mice. Our results showed that the silent synapses were recruited in naive and observer mice, but not in demonstrator mice (Figures 5C,F). Not all slices could successfully recruit silent channels after LTP induction. The map of spatial properties of recruited silent channels was obtained for three slices from three naive mice and for six slices from six observer mice. A majority of the recruited channels appeared at the edge of the area of activated channels (Figures 5D,E).

Analysis of the number and fEPSP amplitude of recruited channels showed that the average fEPSP amplitude of all recruited channels increased significantly in the superficial layers of the aIC of observer mice compared with naive mice [Amplitude: 11.506 ± 3.009 μV of baseline in naïve, 18.504 ± 3.519 μV in observer and 0.799 ± 1.479 μV of baseline demonstrator for superficial layer, *t*_(18)_ = 8.351, *p* = 0.0014, compared by unpaired *t*-test with Welch’s correction, observer vs. naïve, naïve *n* = 3 slices of 3 mice and observer *n* = 6 slices of 6 mice] (Figure 5G). However, in the deep layers of the aIC, there were no discernible qualitative differences between observer and naive mice [Amplitude: 13.937 ± 1.833 μV in naïve, 13.411 ± 1.879 μV in the observer, and −0.587 ± 1.425 μV in the demonstrator for superficial and deep layer, *t*_(18)_ = 0.983, *p* = 0.339, unpaired *t*-test] (Figure 5I). In the observer mice, the number of recruited channels in the superficial layers was, on average, similar to that in the naive mice [Channel: 1.333 ± 0.333 channels in naive and 1.800 ± 0.374 channels in observer, *t*_(6)_ = 0.262, *p* = 0.802, unpaired *t*-test] (Figure 5H). However, in the deep layers of the aIC, fewer channels were recruited in the observer than in the naive mice [Channel: 3.667 ± 0.667 channels in naive and 1.800 ± 0.374 channels in observer, *t*_(6)_ = 2.678, *p* = 0.037, unpaired *t*-test] (Figure 5J). The results, which include both the recruited fEPSP amplitude and the number of recruited channels, suggest that psychological distress elicits strongly recruited responses in the superficial layers but produces silencing in the deep layers of the aIC. Nevertheless, we could not find recruited responses during physiological stress after LTP induction.

### Altered the Cortical Long-Term Depression in the Anterior Insular Cortex of Observer and Demonstrator

**FIGURE 6:**
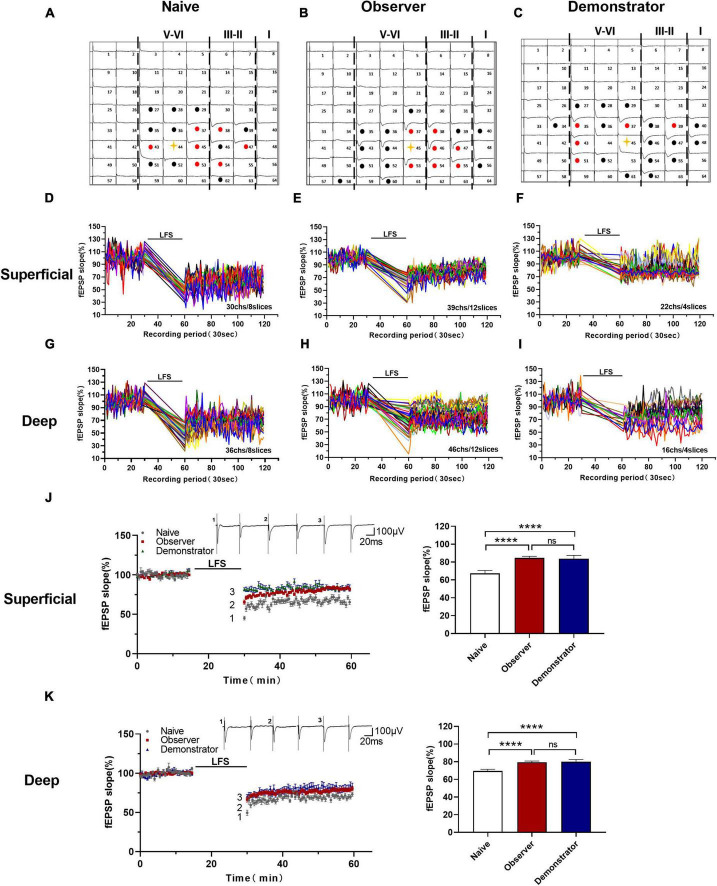
Altered the cortical LTD in the aIC of observer and demonstrator mice. **(A–C)** Samples of an overview of multisite synaptic responses recorded at baseline (black) and showing LTD after LFS (red) in naïve **(A)**, observer **(B)**, and demonstrator mice **(C)**, respectively. The flash denotes the stimulated channel, red- and black-filled circles mark all activated channels, vertical lines demarcate different layers; **(D–I)** All channels with LTD in the superficial and deep layers of naïve, observer, and demonstrator mice. **(D)** 30 channels of 8 slices with LTD in the superficial layers of aIC in naïve mice. **(E)** 39 channels of 12 slices with LTD in the superficial layers of aIC in observer mice. **(F)** 22 channels of 4 slices with LTD in the superficial layers of aIC in demonstrator mice. **(G)** 36 channels of 8 slices with LTD in the deep layers of aIC in naïve mice. **(H)** 46 channels of 12 slices with LTD in the deep layers of aIC in observer mice. **(I)** 16 channels of 4 slices with LTD in the deep layers of aIC in demonstrator mice. **(J,K)** Left: Time course of averaged fEPSP slope of all active channels from the superficial **(J)** and deep layers **(K)** of aIC in naïve, observer, and demonstrator mice. The line indicates the time of LFS application in the deep layer V/VI. Right: the average slope and of all active channels within the last 10 min recording in the superficial **(J)** and deep layers **(K)** of aIC. Data are shown as mean ± SEM (Naïve *n* = 8 slices of 7 mice, observer *n* = 12 slices of 8 mice, and demonstrator *n* = 4 slices of 4 mice) and compared by one-way ANOVA analysis followed by unpaired *t*-test, ****p < 0.0001, vs. naïve.

Insular cortex synapses are characterized by biphasic plasticity. In addition to LTP, LTD is another form of synaptic plasticity that plays a role in various brain functions and is lost in the anterior cingulate cortex after amputation (severe physiological stress) (Bliss and Cooke, 2011; Kang et al., 2012; Zhuo, 2016). To assess the difference between psychological and physiological stress-related changes in LTD induction, we used an LFS protocol (1 Hz, for 15 min) to induce long-lasting depression in the aIC of naive, observer, and demonstrator mice in a temporal–spatial manner (Figures 6A–C). We then compared LTD differences in naive, observer, and demonstrator mice. Activated channels in 8 slices from 7 naive mice, 12 slices from 8 observer mice, and 4 slices from 4 demonstrator mice were observed. In the naive mice, we found 30 channels in the superficial layers and 36 channels in the deep layers showing LTD (Figures 6D,G); in the observer mice, we found 39 channels in the superficial layers and 46 channels in the deep layers showing LTD (Figures 6E,H), and 22 channels in the superficial layers and 16 channels in the deep layers were recorded in demonstrator mice (Figures 6F,I). The superficial and deep layers of the aIC in the observer group showed a higher slope than in the naive group [Slope: 67.375 ± 3.274% of baseline in naïve, 84.644 ± 1.950% in observer for superficial layers, *t*_(38)_ = 21.520, *p* < 0.0001, 69.583 ± 2.747% in naïve and 78.943 ± 2.706% for deep layers, *t*_(40)_ = 16.230, *p* < 0.0001, unpaired *t*-test, vs. naïve *n* = 8 slices of 7 mice, observer *n* = 12 slices of 8 mice]. However, these were not significantly different when compared with those of demonstrator mice [Slope: 83.772 ± 3.011% of baseline for superficial layers, *t*_(38)_ = 0.959, *p* = 0.344, 80.480 ± 3.593% of baseline for deep layers, *t*_(43)_ = 0.294, *p* = 0.073, observer vs. demonstrator, unpaired *t*-test, *n* = 4 slices of 4 mice] (Figures 6J,K).

**FIGURE 7:**
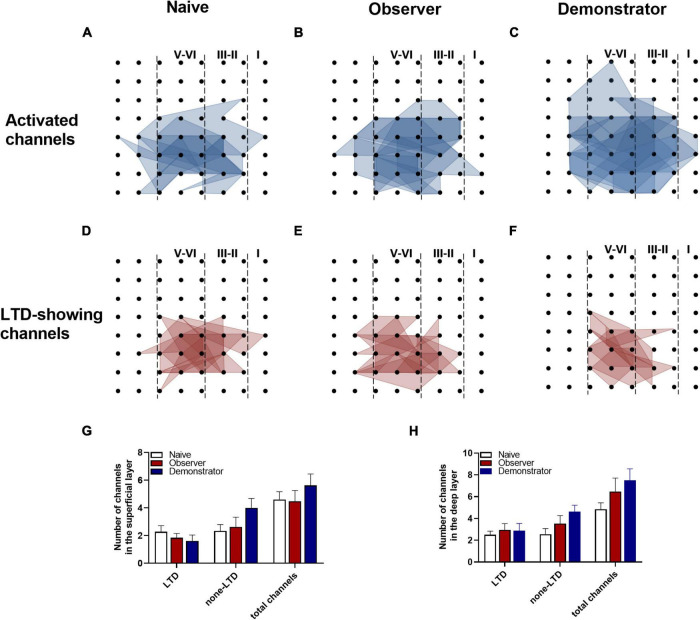
Spatial representation of aIC LTD. **(A–F)** The polygonal diagram of activated (blue) and LTD-showing (red) channels within aIC after LFS in naïve **(A,D)**, observer **(B,E)**, and demonstrator mice **(C,F)**; **(G,H)** Average number of channels of naïve, observer, and demonstrator mice in the superficial **(G)** and deep layers **(H)**. Data are shown as mean ± SEM (Naïve *n* = 8 slices of 7 mice, observer *n* = 12 slices of 8 mice, and demonstrator *n* = 4 slices of 4 mice) and compared by two-way ANOVA analysis followed by unpaired *t*-test, vs. naïve.

Furthermore, we estimated the number of activated channels showing LTD mapped in the spatially characteristic manner of the aIC. Among all groups, the probability of observing LTD was highest in the channels around the stimulation site, and the surrounding channels in layers II/III and V also frequently showed LTD. Not every activated channel transitioned to LTD (Figures 7A–F). Neither the total number of activated channels nor the number of channels with LTD differed on average between naive and observer mice in the superficial and deep layers from each slice of the aIC [Superficial layer: 2.278 ± 0.441 channels with LTD and 2.333 ± 0.464 channels with none-LTP in naive, 1.842 ± 0.308 channels with LTD and 2.632 ± 0.698 channels with none-LTD in the observer, 1.625 ± 0.420 channels with LTD and 4.000 ± 0.681 channels with none-LTD in the demonstrator. LTD channel: *t*_(35)_ = 0.816, *p* = 0.419, naive vs. observer; *t*_(25)_ = 0.396, *p* = 0.696, demonstrator vs. observer; *t*_(24)_ = 0.903, *p* = 0.376, demonstrator vs. naïve. None-LTP channels: *t*_(35)_ = 0.352, *p* = 0.727, naive vs. observer, *t*_(25)_ = 1.170, *p* = 0.253, demonstrator vs. observer; *t*_(24)_ = 2.003, *p* = 0.057, demonstrator vs. naïve. Deep layer: 2.500 ± 0.336 channels with LTP and 2.556 ± 0.519 channels with none-LTP in naive, 2.947 ± 0.585 channels with LTP and 3.526 ± 0.739 channels with none-LTP in observer, 2.875 ± 0.666 channels with LTP and 4.625 ± 0.596 channels with none-LTP in demonstrator. LTD channel: *t*_(35)_ = 0.654, *p* = 0.518, naive vs. observer; *t*_(25)_ = 0.072, *p* = 0.943, demonstrator vs. observer; *t*_(24)_ = 0.561, *p* = 0.580, demonstrator vs. naïve. None-LTP channels: *t*_(35)_ = 1.064, *p* = 0.295, naive vs. observer, *t*_(25)_ = 0.907, *p* = 0.373, demonstrator vs. observer; *t*_(24)_ = 2.359, *p* = 0.068, demonstrator vs. naïve, unpaired *t*-test, naïve *n* = 8 slices of 7 mice, observer *n* = 12 slices of 8 mice and demonstrator *n* = 4 slices of 4 mice] (Figures 7G,H). These data suggest that observational distress and shock similarly alter LTD in both superficial and deep layers of the aIC.

### Functional Synapses Are Silent After the Induction of Long-Term Depression in the Anterior Insular Cortex

**FIGURE 8:**
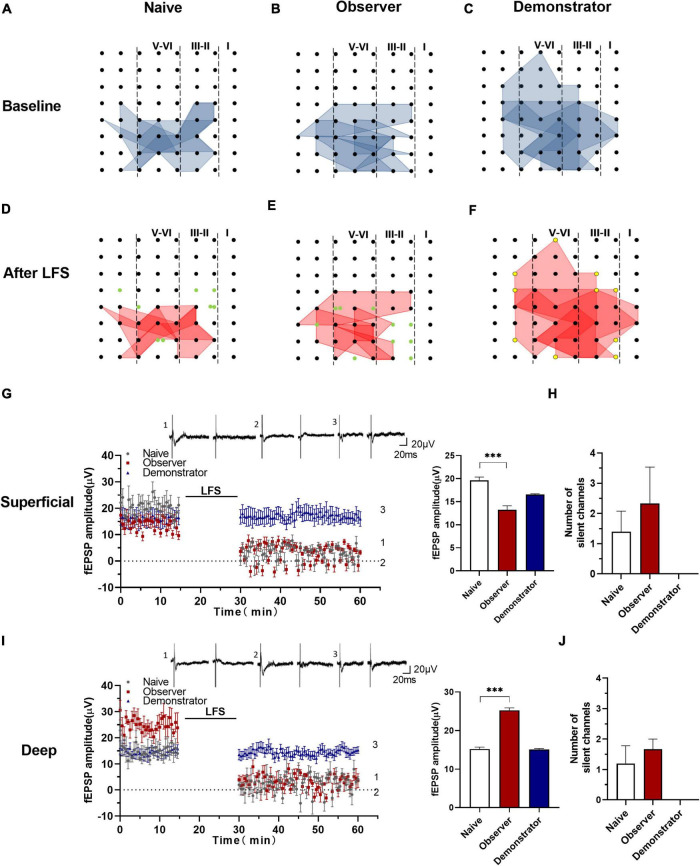
Silencing responses after the induction of LTD in aIC. **(A–F)** The network propagation of synaptic responses in aIC of naïve **(A,D)**, observer **(B,E)**, and demonstrator mice **(C,F)**; Basal-activated areas (blue) and recruited areas (red) and the silent channels are shown as green, the channels at the edge of the activated channels in demonstrator mice are shown as yellow spot; **(G,I)** Left: Time course of averaged fEPSP amplitude of silent channels from the superficial **(G)** and deep layers **(I)** in naïve and observer mice and the no responses channels in demonstrator mice. The line indicates the time of LFS application in the deep layer V/VI; Right: the average fEPSP amplitude of the channels within the last 10 min baseline recording in the superficial **(G)** and deep layers **(I)**. **(H,J)** Average number of silent channels of naïve, observer, and demonstrator mice in the superficial **(H)** and deep layers **(J)**. Data are shown as mean ± SEM (naïve *n* = 4 slices of 4 mice, observer *n* = 4 slices of 4 mice, and demonstrator *n* = 4 slices of 4 mice) and compared by unpaired *t*-test with Welch’s correction, ^***^ *p* < 0.001, vs. naïve.

Previous studies suggest that functional ensembles are strengthened, the total number of excitatory synapses would decrease, which can be transformed into silent synapses at equilibrium by α-amino-3-hydroxy-5-methyl-4-isoxazolepropionic acid (AMPA) receptor mediation (Xiao et al., 2004; Koch and Ullian, 2010; Shukla et al., 2017). In this experiment, some activated channels were converted to silent channels after LFS in naïve and observer, not in demonstrator mice (Figures 8C,F). From the point of view of the temporal–spatial distribution map (Figures 8D,E), the silent channels appeared in the layers II/III and V, at the edge of the activated areas. The fEPSP amplitudes of the activated channels in the superficial and deep layers decreased to approximately 0 μV after LFS. At baseline, fEPSP amplitudes in the superficial layers were lower in the observer than in naive mice [Amplitude in baseline: 19.636 ± 3.118 μV in naïve, 13.249 ± 2.252 μV in the observer, and 16.545 ± 2.358 μV of baseline demonstrator for superficial layer, *t*_(18)_ = 5.716, *p* < 0.001, compared by unpaired *t*-test with Welch’s correction, observer vs. naïve, naïve *n* = 8 slices of 7 mice, observer *n* = 12 slices of 8 mice] (Figure 8G), but higher in the deep layers [Amplitude in baseline: 15.215 ± 3.312 μV in naïve, 25.254 ± 4.164 μV in observer, and 15.076 ± 1.642 μV of baseline demonstrator for superficial layer, *t*_(18)_ = 12.530, *p* < 0.001, compared by unpaired *t*-test with Welch’s correction, observer vs. naïve] (Figure 8I). Regardless of whether in the superficial or deep layers, the number of the silent channels that occurred in the aIC on average was not statistically different between observer and naive mice [Superficial layer: 1.400 ± 0.678 channels in naïve, 2.333 ± 1.202 channels in observer, *t*_(6)_ = 0.741, *p* = 0.487; Deep layer: 1.200 ± 0.583 channels in naïve, 1.667 ± 0.333 channels in observer, *t*_(6)_ = 0.573, *p* = 0.588, unpaired *t*-test] (Figures 8H,J). Compared with naive mice, there was a layer-related difference in silent responses at baseline after psychological stress. There was no silent response during physiological stress after LFS.

## Discussion

To better describe the differential effects of physiological and psychological stress on animals, we developed an observational fear model in which the observer mice acquired fear by social transmission from the shocked demonstrator mice. This model could reflect salient aversive or arousal properties that psychological distress shares with physiological stress. In addition, the observed fear response in the absence of prior shocks might be due to social transmission rather than an evoked memory of one’s shock experience.

The IC integrates social affective stimuli, acting as a link between multimodal sensory inputs and emotional, executive, and social circuits in the limbic system (Gogolla, 2017). The aIC integrates top–down and bottom–up information in the brain, that is consistently activated during feeling and observed disgust (Calder et al., 2000; Wicker et al., 2003). Selectively photoactivation of GABAergic neurons of the aIC remarkably promoted cued fear extinction and alleviated anxiety in a PSTD mouse model (Shi et al., 2020). Behaviors are likely correlated with electrophysiological activities in the brain, including synaptic spontaneous discharge, presynaptic transmitter release, LTP, and LTD. However, only a few efforts have been made to elucidate the difference between psychological and physiological stress through synaptic transmission and plasticity in the aIC at the physiological level. In addition, neurons in different layers of IC are thought to receive different neuronal inputs (Zhuo, 2016). The pyramidal neurons in the superficial layers receive emotional signals and visceral inputs from the medial thalamus, whereas the neurons in the deep layers project toward subcortical structures to provide descending sensory control (Zhuo, 2008; Lu et al., 2016; Gogolla, 2017; Watson and Puelles, 2017). Therefore, we compared the electrophysiological changes of the different layers in the aIC after the two types of stress.

Miao et al. (2019) reported that *N*-methyl-D-aspartic acid (NMDA) receptor-independent presynaptic LTP (pre-LTP) could occur in superficial and deep layers of the IC. Our previous study also indicated that anxiety stimuli resulted in the selective occlusion of pre-LTP, and characterized a form of pre-LTP that requires kainate receptors in neurons of the agranular insular cortex (Shi et al., 2018). And it may constitute a synaptic mechanism by which anxiety regions interact (Koga et al., 2015). Like pre-LTP, PPR is commonly used to measure presynaptic function as well. Our results showed that basal glutamatergic synaptic transmission was not altered by stress. We also found that PPR in slices of observer and demonstrator mice at 50-ms intervals was significantly lower than that of naive mice in the superficial layers but not in the deep layers of the aIC. It appears that both the social–psychological stress and the physiological properties of the stress experience enable NMDA receptor–independent presynaptic plasticity in the superficial layers of the aIC.

Excitatory synapses in the IC are highly plastic. TBS elicits protein synthesis–dependent LTP in neighboring regions, including the superficial and deep layers of the IC (Liu et al., 2013a). In the present work, we used LTP to determine whether synaptic responses in the aIC are enhanced after observational fear or shock, and show that less potentiation is induced after observational fear and no potentiation is induced after shock. Compared with previous experiments in the IC (Qiu et al., 2013), LTP was partially reduced by administration of NVP-AMM077 (GluN2A receptor antagonist) or Ro 25-6981 (GluN2B receptor antagonist). LTP is weaker in observer mice, which might be affected by psychological stress. LTP in demonstrator mice approaches the AP-5-blocked LTP in potentiation in the IC, consistent with our previous study (Shi et al., 2018). The synaptic responses recorded by the MED64 system are due to local synaptic networks rather than general field responses of the same cell population (Kang et al., 2012). Spatial analysis of LTP distribution showed that part of the activated channel undergoes LTP, and the spread of channels with LTP in observer mice is not significantly different from that of naive mice but is wider than that of demonstrator mice. Furthermore, there was no apparent difference among layers in the number of channels showing LTP between observer and demonstrator mice. Because stress has been reported to affect memory formation, glucocorticoids affect NMDA-dependent synaptic plasticity, which is correlated with cognitive memories, and it may enhance emotional memories (Quirarte et al., 1997; Maggio and Segal, 2012). Similarly, in the aIC, physiological stress may form stronger emotional memories than psychological stress by damaging cognitive memories.

Long-term depression is another kind of synaptic plasticity, which is enduring changes in synaptic strength, as a cellular model of information storage and process in the CNS (Martin et al., 2000), and which is used to assess the stress in adult male mice (Lee et al., 2021). In our work, we found a weaker cortical LTD in the superficial and deep layers of the aIC in observer and demonstrator mice, but there was no significant difference between them. The number of LTD channels in the superficial layers of demonstrator mice tended to decrease. We found no layer-related difference in fEPSP slope and the number of LTD channels or total activated channels. Although there is no significant difference in neuronal plasticity in the aIC between physiological and psychological stress, using LTD as a readout of the synaptic consequences of stress, psychological stress triggers synaptic plasticity in the aIC in the same way as physiological stress.

An interesting finding is that LTP induction elicited recruited responses in the aIC of naive and observer mice, but not in demonstrator mice. It is hypothesized that altered synaptic responses contribute to fear conditioning (Steenland et al., 2012). The recruitment of synaptic responses could be caused by enhancement of presynaptic glutamate release, silent synapses, or postsynaptic trafficking of AMPAR (Chen et al., 2014a). Given the differences in PPF, LTP, and LTD between naive, observer, and demonstrator mice, there is insufficient evidence to understand the reason for the observed synaptic responses. In our experiment, no synaptic responses were observed in the aIC after physiological stress, possibly because AMPAR trafficking was not induced. It has been found that new silent synapses including novo synaptogenesis and the removal from regular synapses are formed in the adult brain after exposure to injury (Lo et al., 2011), stress (Suvrathan et al., 2014), or observational fear (Ito et al., 2015). LTD-inducing stimulation of functional synapses resulted in AMPA-silent synapses, which could subsequently be unsilenced by renewed LTP-inducing stimulation (Montgomery et al., 2001). Synapses are recruited and re-silenced during memory destabilization after memory reactivation, and then mature again when memory reconsolidates (Wright et al., 2020). Based on the observed variation, there are layer-related differences in the response to observational fear in the aIC. Presumably, observational distress (psychological stress) generates silent synapses in the deep layers but converts them to active ones in the superficial layers. This suggests that the superficial and deep layers of the aIC are involved in different ways in the reactivation of memory after psychological stress.

In summary, we used a multielectrode recording approach to investigate the spatial distribution and induction of LTP and LTD in the aIC of an observational fear learning mouse model. We found that both psychological and physiological stress enhanced presynaptic transmission in mice. The occurrence of LTP after psychological stress is higher than that after physiological stress. We observed no significant differences in LTD between psychological and physiological stress, although we cannot rule out a possible difference in other forms of LTD. In addition, we found that recruited responses after TBS show layer-related differences as a consequence of psychological stress but not physiological stress. These findings shed light on the regulation of aIC plasticity and stress-induced brain dysfunction following psychological or physiological stress.

## Data Availability Statement

The original contributions presented in the study are included in the article/supplementary material, further inquiries can be directed to the corresponding authors.

## Ethics Statement

The animal study was reviewed and approved by the Institute Animal Care and Use Committee (IACUC) of the National Beijing Center for Drug Safety Evaluation and Research (NBCDSER) (No. 2018-030).

## Author Contributions

TS, YF, and WZ were involved in designing the study. WS carried out all experiments, analyzed the data, and wrote the manuscript. TS helped to revise the manuscript. YF helped to do the behavioral testing. TS and WZ participated in revising the manuscript and approving the submitted version. All authors have read and agreed to the published version of the manuscript.

## Conflict of Interest

The authors declare that the research was conducted in the absence of any commercial or financial relationships that could be construed as a potential conflict of interest.

## Publisher’s Note

All claims expressed in this article are solely those of the authors and do not necessarily represent those of their affiliated organizations, or those of the publisher, the editors and the reviewers. Any product that may be evaluated in this article, or claim that may be made by its manufacturer, is not guaranteed or endorsed by the publisher.
